# Isolation, identification, and genomic analysis of bacteriophage GJKY-A for the prevention and treatment of citrus canker

**DOI:** 10.3389/fmicb.2025.1703194

**Published:** 2025-12-12

**Authors:** Yang Wu, Zexia Li, Kexin Li, Weiwei Li, Mingxue Chang, Dingkun Liu, Songjia Hu, Li He, Huimin Sun

**Affiliations:** School of Life Sciences, Jinggangshan University, Ji’an, China

**Keywords:** *Xanthomonas citri* subsp. *citri*, bacteriophage, phage control, biological characteristics, phage genome analysis

## Abstract

**Introduction:**

Citrus canker, caused by *Xanthomonas citri* subsp. citri (Xcc), leads to severe economic losses in global citrus production. Conventional chemical control strategies are increasingly hindered by bacterial resistance and environmental concerns. Bacteriophages offer a sustainable alternative for plant disease management.

**Methods:**

This study isolated bacteriophages from wastewater using a double-layer plate method, and conducted biological characterization and genomic analysis on the isolated bacteriophages.

**Results:**

We report the isolation and characterization of a phage, GJKY-A, from sewage effluent targeting Xcc. Transmission electron microscopy revealed an polyhedral head (~90 nm) and a long, flexible non-contractile tail (~150 nm). One-step growth analysis showed a latent period of 20 min and a burst size of ~84 plaque-forming units per cell. GJKY-A displayed high host specificity and remained stable across 40–50 °C and pH 3–12. Genomic sequencing identified a 62,551 bp double-stranded DNA genome with 44.81% GC content. Analysis of the taxonomic status of the phage shows that phage GJKY-A is closely related to phages of the genus *Stenotrophomonas*. Functional assays demonstrated efficient inhibition of host growth *in vitro* and suppression of citrus canker symptoms in ex vivo leaf assays.

**Discussion:**

These findings establish GJKY-A as a promising biocontrol candidate and provide a foundation for developing safe, eco-friendly strategies for citrus canker management.

## 1 Introduction

Citrus canker, caused by the bacterial pathogen *Xanthomonas citri* subsp. *citri* (Xcc; synonyms: *X. campestris* pv. *citri* or *X. axonopodis* pv. *citri*), poses a major threat to the global citrus industry, requiring quarantine and destruction of crops (Yao et al., 2015). The disease is highly transmissible and notoriously difficult to manage, with no definitive eradication strategy established to date (Savietto et al., 2018). In recent years, the rapid expansion of large-scale and intensive citrus cultivation has markedly increased the risk of outbreaks, with citrus canker shifting from sporadic occurrences to epidemic proportions. Severe infections result in extensive leaf defoliation, premature fruit drop, and even host plant death (Ali et al., 2023). Infected fruits exhibit pronounced quality deterioration, while citrus products from quarantined regions face strict domestic and international phytosanitary restrictions, significantly hindering the sustainable development of citrus industries across multiple regions in China.

Current management relies primarily on agronomic practices and the extensive use of bactericides throughout the citrus growth cycle. However, insufficient grower awareness of varietal susceptibility, epidemiological patterns, bactericide properties, application timing, and climatic influences often compromises disease control (Behlau et al., 2017). Consequently, the rotation or combination of bactericides has become routine, with emphasis on varying bactericides with alternating modes of action to reduce the risks of resistance and soil contamination associated with the prolonged use of a single formulation. These limitations underscore the urgent need for novel, sustainable control agents.

Bacteriophages, as the most abundant biological entities in nature, can be readily isolated from diverse environments and lyse target pathogens with high specificity, including multidrug-resistant bacteria, without disrupting non-target microbial populations (O'Flaherty et al., 2009). As bacterial viruses, bacteriophages are considered green and safe biocontrol agents, as they leave no chemical residues and also reduce the risk of the development of drug resistance (Bao et al., 2011). These features highlight the potential of bacteriophages as potential next-generation biopesticides, attracting extensive research attention in recent years (Salmond and Fineran, 2015; Balogh et al., 2010). Substantial progress has been made in the development and application of bacteriophages in the agricultural field. For example, the team led by Shen Qirong from Nanjing Agricultural University systematically established a national library of obligate bacteriophages targeting *Ralstonia solanacearum*. Specifically, they used a “cocktail” combination formula to reduce the number of pathogenic bacteria and restore the balance of soil microbial communities, which ultimately reduced the incidence of bacterial wilt in tomatoes by 80% (Wang et al., 2019). In 2018, a bacteriophage pesticide product launched by Omnilytics was approved by the U. S. Environmental Protection Agency for commercial purposes.

The isolation of phages against Xcc (the causal agent of citrus canker) has also attracted increasing attention recently. Cp1 and Cp2 are among the earliest discovered phages of Xcc, showing effectiveness against multiple strains; therefore, these phages have largely been used in phage typing and biological control research (Yao et al., 2015). In addition, the phage XacN1 showed strong lytic activity against Xcc and is considered another candidate for phage therapy (Yoshikawa et al., 2018). Bacteriophage MK21 can lyse Xcc and shows high stability under various environmental conditions (Moon et al., 2024). Therefore, Isolating bacteriophages of *Xanthomonas citri* subsp. *citri* is of great significance for the prevention and control of citrus canker. To expand this research, we used the citrus canker pathogen Xcc as the host bacterium for phage screening. A virulent lytic phage was successfully isolated from wastewater, and its biological properties and genomic features were systematically characterized. This study therefore provides a foundation for the development of phage-based biocontrol agents against citrus canker.

## 2 Materials and methods

### 2.1 Host strain culture and identification

The host bacterium used in this study was *Xanthomonas citri strain 3,213* (NR_104964.1), which provided by the National Navel Orange Engineering Research Center. To obtain an enrichment culture of *Xcc 3,213*, 500 μL of the host bacterial solution was added to a centrifuge tube containing 10 mL of Luria–Bertani (LB) liquid medium and then placed in an incubator for shaking culture at 28 °C for 24 h.

The bacterium was identified by sequencing. A fragment of 16S rDNA was amplified using polymerase chain reaction (PCR) in a 50 μL reaction mixture containing 48 μL of 1 × Fast Mix, 0.5 μL each of the forward primer 27F (5′–AACTCCAGCACATACGGGTC–3′) and reverse primer 1492R (5′–TACGGYTACCTTGTTACGACTT–3′), and 1 μL of template DNA (10–50 ng). The PCR program consisted of an initial denaturation step at 95 °C for 5 min, followed by 35 cycles of denaturation at 95 °C for 30s, annealing at 55 °C for 30s, and extension at 72 °C for 1 min, with a final extension at 72 °C for 10 min. The PCR products were separated by electrophoresis on agarose gels at 120 V for 15 min and visualized under ultraviolet illumination. Purified PCR products were sequenced commercially by Sangon Biotech (Shanghai) Co., Ltd. for confirmation.

### 2.2 Bacteriophage isolation, purification, and identification

#### 2.2.1 Phage isolation

Wastewater samples were obtained from the Ji’an City Wastewater Treatment Plant (Jiangxi Province, China) and screened for phages according to previously described isolation protocols (Gao et al., 2024). The samples were first filtered through double-layer filter paper and centrifuged at 4,000 rpm for 10 min at 28 °C. The supernatant was subsequently passed through a 0.22-μm membrane filter. For phage enrichment, 10 mL of the filtrate was mixed with an equal volume of 2 × LB broth (with calcium chloride added to obtain a total calcium ion concentration of 2.07 mmol/L) and inoculated with 1 mL of the host bacterium [optical density at 600 nm (OD600) of 0.6]. The mixture was incubated overnight at 28 °C with shaking, followed by centrifugation at 4,000 rpm for 10 min. The supernatant was again filtered through a 0.22-μm membrane filter and stored for further use.

To detect the lytic activity of the isolates, 200 μL each of the phage-containing supernatant and host bacterial suspension (OD600 = 0.6) were mixed with 5 mL of molten semi-solid LB medium (0.75% agar, 50 °C). The mixture was gently combined and overlaid onto solid LB agar plates. After allowing the top layer to solidify for 5 min, the plates were air-dried in a laminar flow cabinet and incubated at 28 °C for 24 h. Plaque formation was subsequently examined.

#### 2.2.2 Phage purification and concentration

Individual plaques were picked using sterile pipette tips and transferred into 1 mL of sterile sodium chloride–magnesium sulfate buffer (SM buffer), followed by gentle shaking for 2 h to release phage particles. The resulting suspension was serially diluted and 0.1 mL of the dilution was mixed with 0.1 mL of host bacterial suspension (OD600 = 0.6). After incubation at room temperature for 15 min, the mixture was added to 5 mL of molten semi-solid LB medium (0.75% agar, 50 °C), mixed thoroughly, and poured onto LB agar plates. The plates were incubated in an inverted position at 28 °C for 24 h. The plaque purification process was repeated until morphologically uniform plaques were obtained.

To determine the phage concentration, solid PEG8000 was added to the purified phage suspension to a final concentration of 100 g/L. The mixture was dissolved thoroughly and incubated on ice for at least 3 h to facilitate phage particle precipitation. The suspension was then centrifuged at 12,000 rpm for 10 min at 4 °C and the supernatant was discarded. The pellet was resuspended in 2 mL of SM buffer, followed by filtration through a 0.22-μm membrane to obtain the concentrated phage.

#### 2.2.3 Phage titer determination

Phage stocks were serially diluted in 10-fold steps. Aliquots of 100 μL of the diluted phage suspension were mixed with 100 μL of the host bacterial culture (OD600 = 0.6) and incubated at room temperature for 15 min. The mixtures were then added to molten semi-solid LB agar (0.75% agar, 50 °C), overlaid onto LB agar plates, and incubated at 28 °C for 24 h. Plaques were subsequently enumerated. Phage titers were calculated as plaque-forming units (PFU) per milliliter using the following formula:

Phage titer (PFU/mL) = average number of plaques × dilution factor × 10.

#### 2.2.4 Transmission electron microscopy (TEM)

Samples were prepared for TEM observation by referring to previously reported methods (Wang et al., 2024). In brief, the purified phage suspension was dropped onto a copper grid and left to react for 10 min. Filter paper was used to absorb the excess liquid on the copper grid from the side. Subsequently, one drop of 2% phosphotungstic acid (pH 7.0) was added to the copper grid. After staining for 10 min, the copper grid was placed on dry filter paper and left to dry naturally. The concentrated phage solution was then observed on a transmission electron microscope (Hangzhou Yanqu Information Technology Co., Ltd.).

### 2.3 Biological characterization of the isolated phage

#### 2.3.1 Host range determination

The bacterial strains used are shown in Table 1. Bacterial lawns were prepared by mixing 200 μL of test bacterial cultures (OD600 = 0.6) with molten semi-solid LB agar (0.75% agar, 50 °C) and pouring the mixture onto LB agar plates. After solidification, 5 μL of the phage lysate was spotted onto the bacterial overlay, air-dried, and incubated at 28 °C for 24 h. Plaque formation at the inoculation site was then assessed. As a negative control, 5 μL of sterile SM buffer was spotted onto parallel bacterial overlays (Minh et al., 2016).

**Table 1:** Host range of bacteriophage GJKY-A.

Species	Infectivity
*Xanthomonas citri strain* 3213	+++
*Escherichia coli* CMCC (B) 44102	−
*Staphylococcus aureus* CMCC (B) 26003	−
*Bacillus subtilis* CMCC (B) 63501	−
*Pseudomonas aeruginosa* CMCC (B) 10104	−

The lysis intensity is indicated by “+++” for strong lysis (clear plaques) and “–” for no lysis.

#### 2.3.2 Optimal multiplicity of infection (MOI) determination

The host bacterial suspension (OD600 = 0.6) was adjusted to a final concentration of 1 × 108 colony-forming units (CFU)/mL, following the method of Lu et al. (2003). Phage suspensions were then added at different MOI ratios (phage-to-bacterium ratios) of 100, 10, 1, 0.1, 0.01, and 0.001. Each mixture was supplemented with LB broth to equalize the total volume across treatments and incubated at 28 °C with shaking at 180 rpm for 4 h. Following incubation, the cultures were centrifuged at 10,000 rpm for 10 min, and the supernatants were filtered through 0.22-μm membranes. Phage titers were subsequently determined, and the MOI yielding the highest phage titer was defined as the optimal MOI.

#### 2.3.3 One-step growth curve assay

Host bacterial suspensions (1 × 10^8^ CFU/mL) were mixed with phage lysates at the optimal MOI and incubated at room temperature for 5 min to allow phage adsorption. The mixtures were centrifuged at 8,000 × g for 1 min and the supernatants were discarded. The pellets were washed twice with LB broth and resuspended in 20 mL of fresh LB medium. The cultures were immediately incubated at 28 °C with vigorous shaking (160 rpm). Samples were collected at 10-min intervals, with three biological replicates per time point, and phage titers were determined to construct the one-step growth curve.

#### 2.3.4 Thermal stability assay

A 600 μL aliquot of the phage lysate (1 × 10^8^ PFU/mL) was incubated in a water bath at different temperatures (40, 50, 60, 70, and 80 °C) for 2 h. Samples were withdrawn every 20 min up to 120 min and residual phage titers were determined using the double-layer agar overlay plaque assay as described above. Phage viability under different temperature conditions was evaluated, and stability curves were generated based on the quantified titers.

#### 2.3.5 pH stability assay

A 100 μL aliquot of phage lysate (1 × 10^8^ PFU/mL) was mixed with 900 μL of SM buffer adjusted to different pH values (pH 2–12) and incubated at 28 °C for 2 h. Residual phage titers were then determined using the double-layer agar overlay plaque assay. Phage viability under varying pH conditions was assessed, and stability curves were plotted based on the quantified titers.

#### 2.3.6 *Ex vivo* leaf inoculation assay

The *ex vivo* inoculation assay was performed with reference to previously described *in vitro* inoculation methods (Mathioudakis et al., 2013), with modifications based on the specific experimental design. Healthy citrus leaves were selected for detached-leaf inoculation to evaluate pathogen virulence and the biocontrol efficacy of the phage. Three experimental groups were established: (A) sterile water control, (B) pathogen inoculation with the bacterial suspension, and (C) phage treatment (bacterial suspension + phage).

Ten microliters of the bacterial suspension (1 × 10^8^ CFU/mL) were added dropwise onto a sterile absorbent filter paper disk and applied to the punctured site on the leaf surface. For Group C, 10 μL of phage lysate (1 × 10^8^ PFU/mL) was pipetted onto the inoculated filter disks. Each treatment was performed in triplicate. The inoculated leaves were maintained in a growth chamber under controlled conditions (28 °C, 60% relative humidity, 12 h light/dark cycle). After 48 h, the filter disks were removed and the leaves were further incubated under the same conditions. Disease progression was monitored and recorded daily.

According to the changes in the disease spots, the leaves on the 3rd, 4th, and 5th days were selected for bacterial counting to verify the antibacterial effect of the phage. The cut diseased leaves were soaked in 70% ethanol for 60 s for disinfection and then rinsed with sterile water 3–4 times. A sterile puncher was used to precisely cut the leaves containing the disease spots with a diameter of 0.6 mm. The leaves were placed in a 2-mL sterile centrifuge tube with 500 μL of sterile phosphate-buffered saline (pH 7.0) and then ground in a tissue grinder. The supernatant was diluted in a 10-fold gradient, and 20 μL of the bacterial solution was pipetted from each dilution, spread onto the medium, and incubated at 28 °C for 24–48 h before calculating the bacterial count.

### 2.4 Phage genome sequencing and annotation

The phage genomic DNA was extracted using a viral DNA extraction kit (Qiagen, Germany) and sequenced by Shanghai Panogenes Biotechnology Co., Ltd. The whole-genome shotgun strategy was adopted to construct libraries with different insert fragments. Next-generation sequencing technology was used to perform paired-end sequencing on these libraries based on the Illumina NovaSeq sequencing platform.

For sequencing data processing and quality assessment, the raw data were saved in paired-end FASTQ format. The quality value (*Q*-value) was calculated as the rounded mapping result of the base reading error rate (p). *Q*- and *p*-values were compared using the Sanger variant calculation method and the calculation method of the early Solexa pipeline (Illumina Genome Analyzer software). According to the results of this analysis, the FASTQ file uses the Illumina 1.8 + version encoding process. By subtracting the offset value of 33 from the ASCII value of all characters, the Q value of the base can be obtained. After conversion, the sequencing error rate was 0.01%.

A5-MiSeq (Coil et al., 2014) and SPAdes (Bankevich et al., 2012) were used for *de novo* assembly of the sequencing data with adapter sequences removed to construct contigs. Sequences were extracted based on the sequencing depth of the assembled sequences, and those with high sequencing depth were compared with sequences in the National Center for Biotechnology Information (NCBI) NT library using the Blastn tool. The viral genome sequences from each assembly result were selected for further analysis. MUMmer (Kurtz et al., 2004) software was used to perform collinearity analysis on the assembly results obtained from the above steps. Pilon software was then used to correct the results to obtain the final viral genome sequence.

The sequence alignment of protein-coding genes was performed using diamond software (Buchfink et al., 2015) based on the NCBI NR database (with relatively high accuracy). The diamond blastp tool was used to align the protein sequences encoded by genes with the protein sequences in the database. The threshold for sequence alignment was set to 1e-6, and the best hit was selected for function determination. Diamond blastp was further used to perform eggNOG annotation on the gene-encoded protein sequences according to the following functional discrimination rules: *E*-value < 1e-6, sequence identity > 30%, and sequence alignment length ≥70% of either protein. The eggNOG number of the best hits was assigned to the corresponding protein-coding gene.

For visualization of the phage genome, the genomic sequence, gene prediction, and non-coding RNA prediction information was integrated into a standard GBK (GenBank) format file. The cgview program was then used to draw the circular map of the genome, which was edited with Photoshop CS.

### 2.5 Phylogenetic analysis

Some key gene products of phages, such as the large subunit of phage terminase, major capsid protein, endolysin, and holin, are typically used to construct phylogenetic trees for phage typing (Ye et al., 2023; Rombouts et al., 2016). We selected the relevant sequences for constructing the phylogenetic tree from the NCBI database, standardized them into FASTA format, and then used the MEGA11 tool to construct the tree using the maximum-likelihood method. The clustering pattern of phages was then analyzed according to the branching pattern as described previously (Zou et al., 2024).

### 2.6 Bacteriophage inhibition of bacterial biofilm formation

Host bacteria were cultured overnight at 28 °C with shaking and diluted in LB broth to a final concentration of 1 × 10^6^ CFU/mL. Aliquots of 100 μL of the bacterial suspension were added to the wells of a 96-well microtiter plate (Hunan Bikeman Holding Co., Ltd.), followed by the addition of 100 μL of the isolated phage (designated GJKY-A) at different MOIs (100, 10, 1, 0.1, 0.01, and 0.001). The plates were incubated at 28 °C for 24 h. Two control groups were established for comparison: (1) bacterial suspension plus 100 μL LB broth and (2) LB broth alone (200 μL).

After incubation, the supernatants were discarded and the wells were washed three times with 200 μL sterile SM buffer. The formed bacterial biofilms were fixed by adding 200 μL methanol per well for 15 min, the methanol was removed, and the plates were air-dried. Each well was stained with 200 μL of 1% (w/v) crystal violet for 30 min, followed by three washes with sterile water. After drying, 200 μL of 95% ethanol was added to solubilize the bound dye for 30 min. Absorbance was measured at 595 nm using a microplate reader to quantify the biofilm biomass. The experiment was performed in triplicate and mean values were calculated.

### 2.7 Data analysis

Data are presented as the mean and standard deviation (SD) of each measurement variable. To visually display the changing trends and differences in the data, Origin 2024 software was used to create plots with corresponding error bars representing the SD.

## 3 Results

### 3.1 Host strain identification

**Figure 1:**
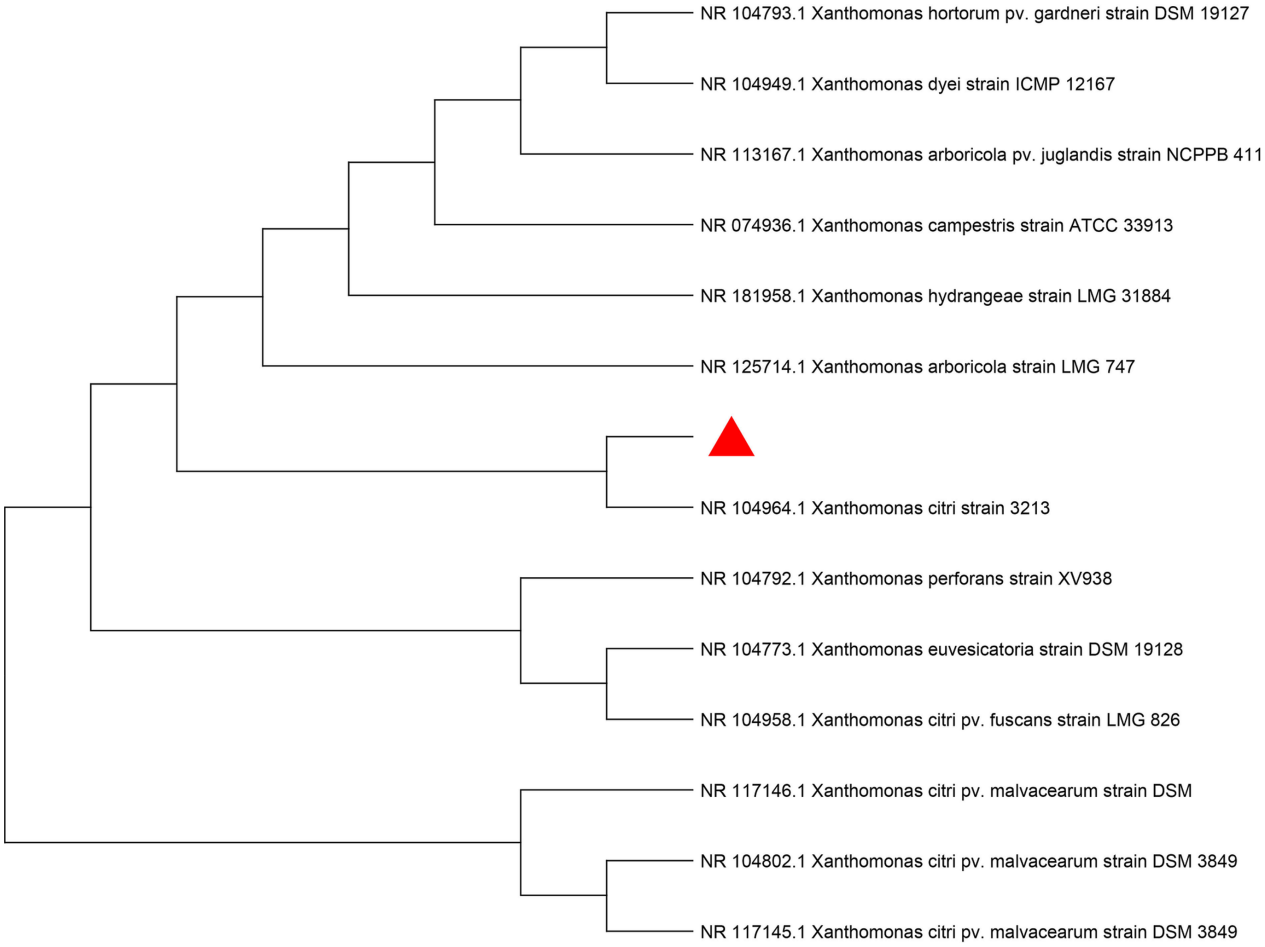
Molecular phylogenetic tree of the host bacterium’s 16S rDNA sequence constructed using the maximum-likelihood (ML) method.

Sequencing of 16S rDNA confirmed 100% sequence query coverage of the tested strain with *Xanthomonas strain 3,213* and the identity reached 99.99%. To further confirm its taxonomic status, similar sequences obtained from the alignment were used to construct a phylogenetic tree in MEGA11 using the maximum-likelihood method. The results showed that the branch between the host bacterium and Xanthomonas strain 3,213 was short, indicating that the genetic difference between them was small. This further confirmed that the host bacterium was Xanthomonas strain 3,213 (Figure 1).

### 3.2 Bacteriophage isolation and characteristics

**Figure 2:**
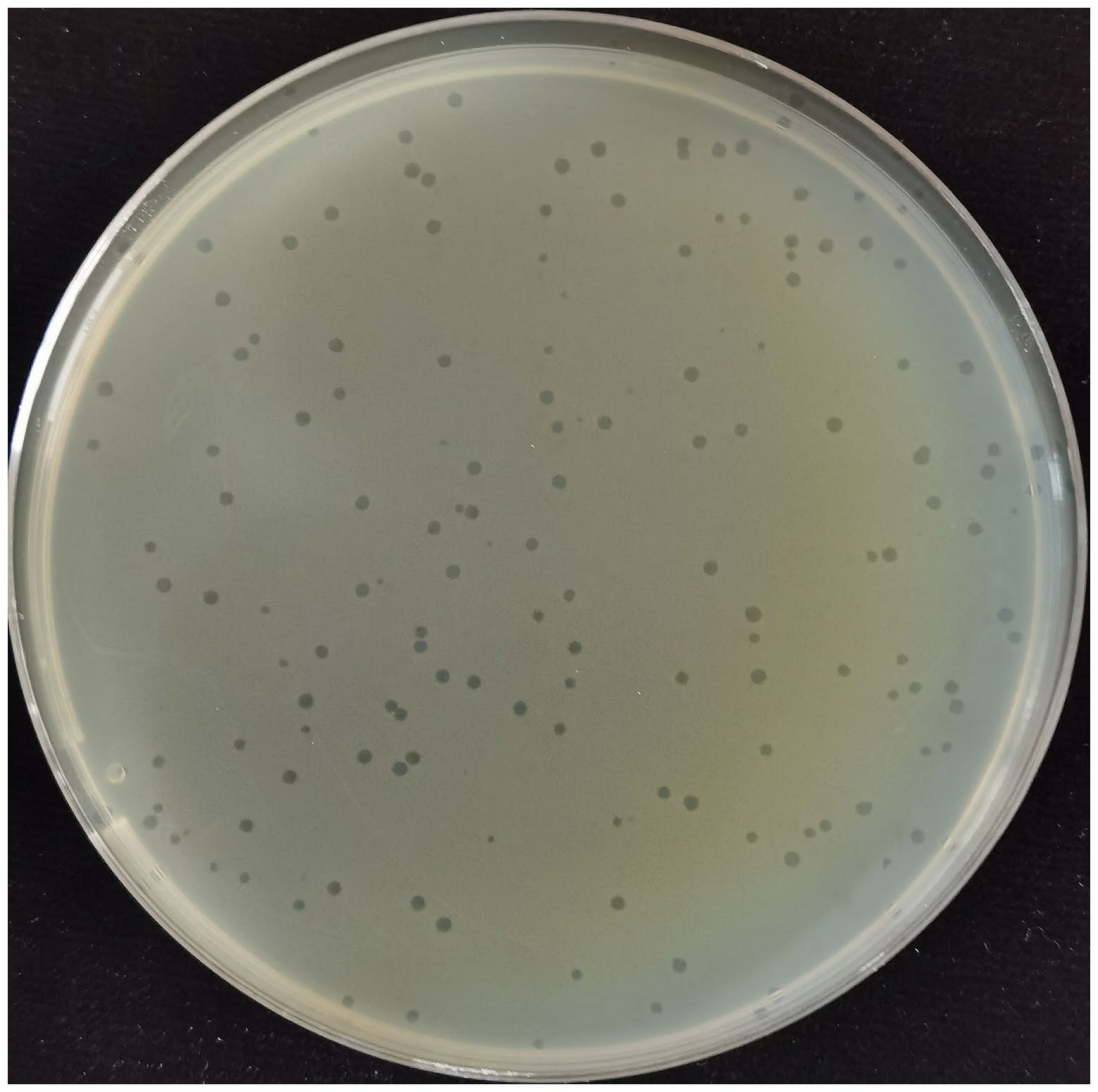
The morphology of plaques produced by bacteriophage GJKY-A on the host bacteria.

Using *Xanthomonas citri strain 3,213* as the host bacterium, a phage was isolated from wastewater samples collected at a municipal treatment plant in Ji’an City, Jiangxi Province, using the double-layer agar method. After incubation for 24 h at 28 °C on lawns of the host bacterium, the phage (designated GJKY-A) produced uniform, circular plaques with well-defined margins and clear lytic halos (Ding et al., 2025) (Figure 2).

#### 3.2.1 Transmission electron microscopy (TEM) of bacteriophage GJKY-A

**Figure 3:**
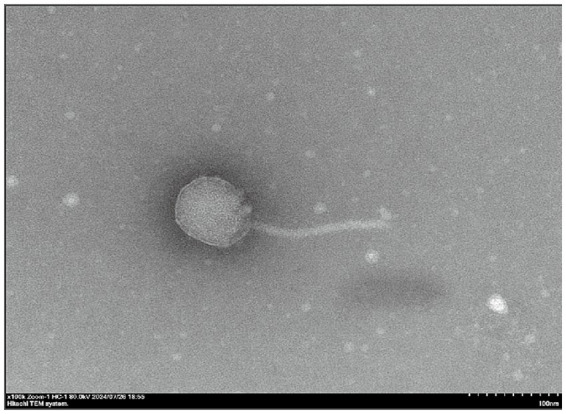
Transmission electron microscopy (TEM) images of bacteriophage GJKY-A. Scale bar = 100 nm. An overall image of bacteriophage GJKY-A. The phage is divided into two parts: the head and the long tail.

TEM observations showed that phage GJKY-A had a polyhedral head with an average diameter of approximately 90 nm and a non-contractile siphovirus with a length of approximately150 nm (Figure 3).

#### 3.2.2 Host range determination

Phage GJKY-A only showed strong lytic activity against its original host Xcc strain, indicating strong specificity for host bacterium (Table 1).

#### 3.2.3 Optimal MOI determination

Phage titers obtained under different MOI conditions are summarized in Table 2. The titer increased proportionally as the MOI was raised from 0.001 to 1, reaching a maximum of 2.05 × 10^10^ PFU/mL^−1^ at MOI = 1. Therefore, the optimal MOI for phage GJKY-A was determined to be 1.

**Table 2:** Optimal multiplicity of infection (MOI) of bacteriophage GJKY-A.

MOI	Phage count (PFU/mL)	Number of host bacteria (CFU/mL)	Phage titer (PFU · mL^−1^)
0.001	1.0 × 10^3^	1.0 × 10^6^	2.8 × 10^7^
0.01	1.0 × 10^4^	1.0 × 10^6^	7.8 × 10^7^
0.1	1.0 × 10^5^	1.0 × 10^6^	4.63 × 10^9^
1	1.0 × 10^6^	1.0 × 10^6^	2.05 × 10^10^
10	1.0 × 10^7^	1.0 × 10^6^	3.7 × 10^9^
100	1.0 × 10^7^	1.0 × 10^5^	3.2 × 10^6^

#### 3.2.4 One step growth curve analysis

**Figure 4:**
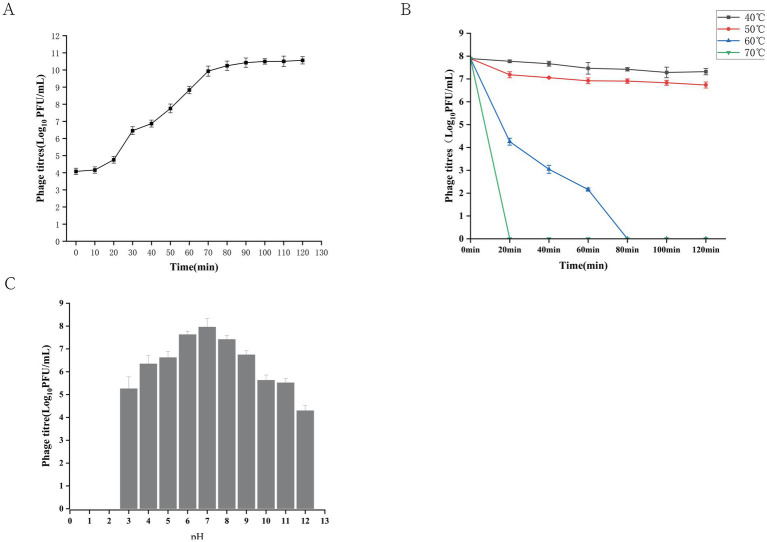
Biological characteristics of bacteriophage GJKY-A. **(A)** One-step growth curve of bacteriophage GJKY-A. **(B)** Thermal stability of bacteriophage GJKY-A (40, 50, 60, and 70 °C). **(C)** pH stability of bacteriophage GJKY-A (range pH 2–12).

The one-step growth curve of phage GJKY-A is presented in Figure 4. No significant increase in phage numbers was observed during the first 20 min post-infection, corresponding to the latent period. A rapid rise in phage titer was recorded between 20 and 70 min, representing the lytic phase. After 70 min, the phage titer stabilized at approximately 3.37 × 10^10^ PFU/mL, indicating entry into the plateau phase. The burst size of phage GJKY-A was estimated to be ~84 PFU per infected cell (Figure 4A).

#### 3.2.5 Thermal stability

Phage GJKY-A remained relatively stable at 40–50 °C for up to 120 min, with only a slight reduction of approximately one order of magnitude in titer. In contrast, exposure to 60 °C for 60 min resulted in complete inactivation of the phage, and phage GJKY-A was fully inactivated at 70 °C (Figure 4B). These results indicated that GJKY-A exhibits moderate thermal tolerance within the range of 40–50 °C, whereas its viability declines sharply at temperatures above 60 °C.

#### 3.2.6 pH stability

Phage GJKY-A remained viable after 120 min of exposure to pH values ranging from 3 to 12 (Figure 4C). The highest titer was observed at pH 7, reaching approximately 1.32 × 10^8^ PFU/mL. In contrast, complete inactivation occurred after 120 min of incubation at pH 2. These results indicated that GJKY-A exhibits tolerance under mildly acidic (pH 3–4) and alkaline (pH 11–12) conditions, demonstrating its ability to withstand both acidic and alkaline environments.

#### 3.2.7 *Ex vivo* biocontrol efficacy

**Figure 5:**
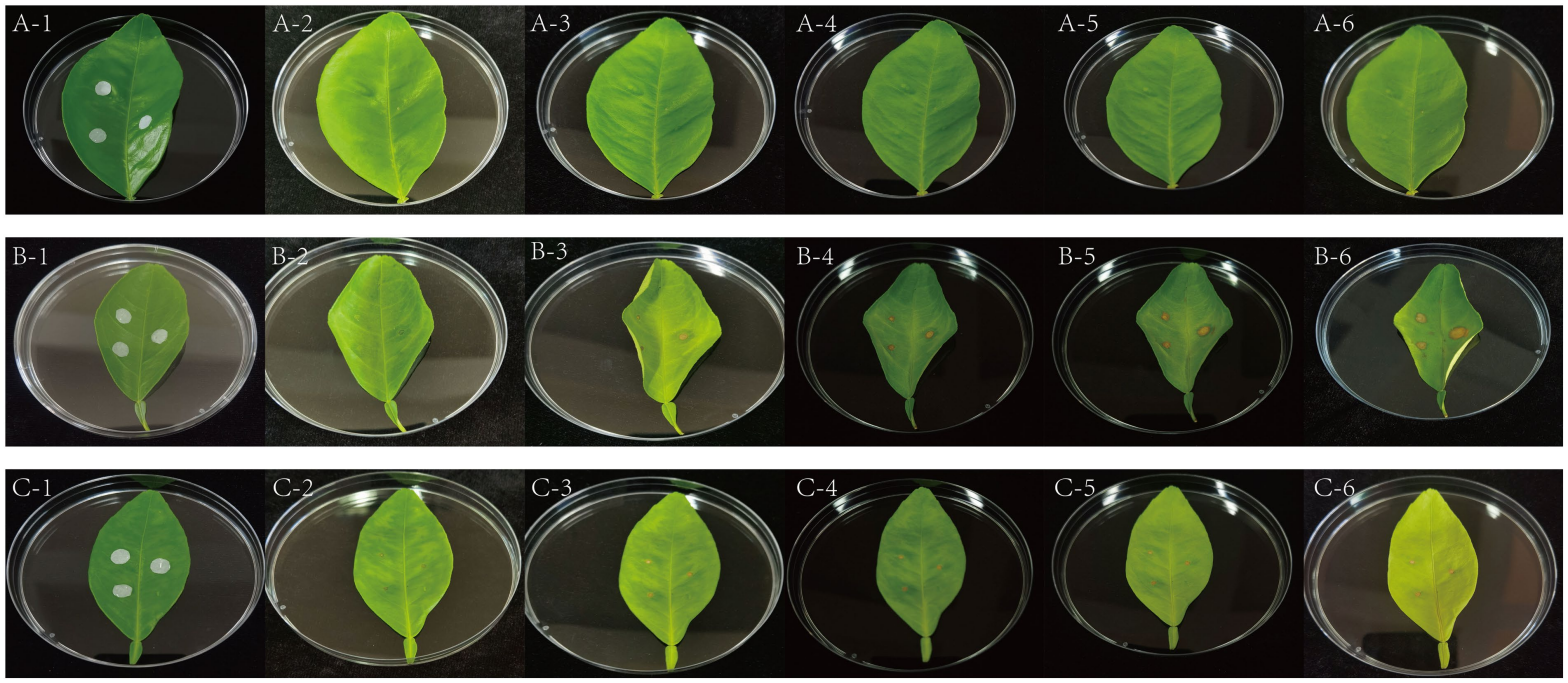
*Ex vivo* inoculation-based biocontrol efficacy of bacteriophage GJKY-A. Group A is the sterile water control group, group B is the host bacteria inoculation group, and group C is the phage treatment group (bacterial suspension + phage). The images depict lesion development at 1, 3, 4, 5, 6, and 7 days post-inoculation, respectively.

As shown in Figure 5 and Tables 3, 4, no suberized canker lesions were observed in the sterile water control group (Group A). However, in the host strain inoculation group (Group B), ulcer spots occurred at the leaf inoculation sites, and the area of the ulcer spots gradually expanded with prolonged incubation time. In contrast, in the phage-treated group (Group C), lesion expansion ceased after phage application on day 3 (C-2) and the expansion of the lesion area stopped, indicating significant suppression of host strain proliferation. After inoculating phages, the expansion of the lesions was significantly inhibited in group C compared with that of group B with only host bacteria inoculation. When the pathogens were inoculated, lesions appeared on the third day after 2 days. Bacterial counts were performed on the leaf lesions on the third, fourth, and fifth days. It was found that on the third day, the bacterial counts in Group B and Group C were approximately the same. In Group B, the bacterial counts continued to increase on the fourth and fifth days. However, after the addition of phages, the bacterial counts in Group C remained approximately the same on the fourth and fifth days, showing no increasing trend. Compared with Group B, the addition of phages could effectively inhibit the growth of the host bacteria in Group C. These results demonstrate that phage GJKY-A exhibits potent bactericidal activity and effectively inhibits the progression of citrus canker lesions on the leaves.

**Table 3:** The area of ulcer spots after in vitro inoculation of leaves.

Training time/day	Lesion diameter of Group B/mm	Lesion diameter of Group C/mm
3d	1.9033 ± 0.0776	2.0667 ± 0.1247
4d	2.2367 ± 0.4000	2.1333 ± 0.1247
5d	3.2000 ± 0.6164	2.1333 ± 0.1247
6d	3.9333 ± 0.8340	2.0333 ± 0.1247
7d	4.9333 ± 1.2499	2.0433 ± 0.0478

The data in the table are presented as the mean ± (standard deviation) SD. The second column of the table shows the changes in the diameter of ulcer lesions in the host bacteria inoculation treatment group (Group B), and the third column shows the changes in the diameter of ulcer lesions in the phage inoculation treatment group (Group C).

**Table 4:** The number of bacteria in the ulcer spots after in vitro inoculation of the leaf blade.

Training time/day	Bacterial concentration in Group B (CFU/mL)	Bacterial concentration in Group C (CFU/mL)
3d	1.75 × 10^3^ ± 0.561 × 10^3^	2.36 × 10^3^ ± 0.772 × 10^3^
4d	2.61 × 10^4^ ± 0.327 × 10^4^	9.03 × 10^3^ ± 0.251 × 10^3^
5d	4.73 × 10^5^ ± 0.673 × 10^5^	9.52 × 10^3^ ± 0.583 × 10^3^

The data in the table are presented as the mean ± standard deviation (SD). The second column in the table shows the changes in the number of bacteria in the ulcer lesions of the host bacteria inoculation treatment group (Group B), and the third column shows the changes in the number of bacteria in the ulcer lesions of the phage inoculation treatment group (Group C).

### 3.3 Genomic features of phage GJKY-A

The genome of phage GJKY-A consists of double-stranded DNA with a total length of 62,551 bp and a GC content of 44.81%. A total of 110 open reading frames (ORFs) were predicted, spanning 58,734 bp (average ORF length: 533.95 bp), which accounted for 93.9% of the genome. Among these, 51 ORFs were assigned putative functions, whereas the remaining are predicted to encode hypothetical proteins (Supplementary Table S1).

**Figure 6:**
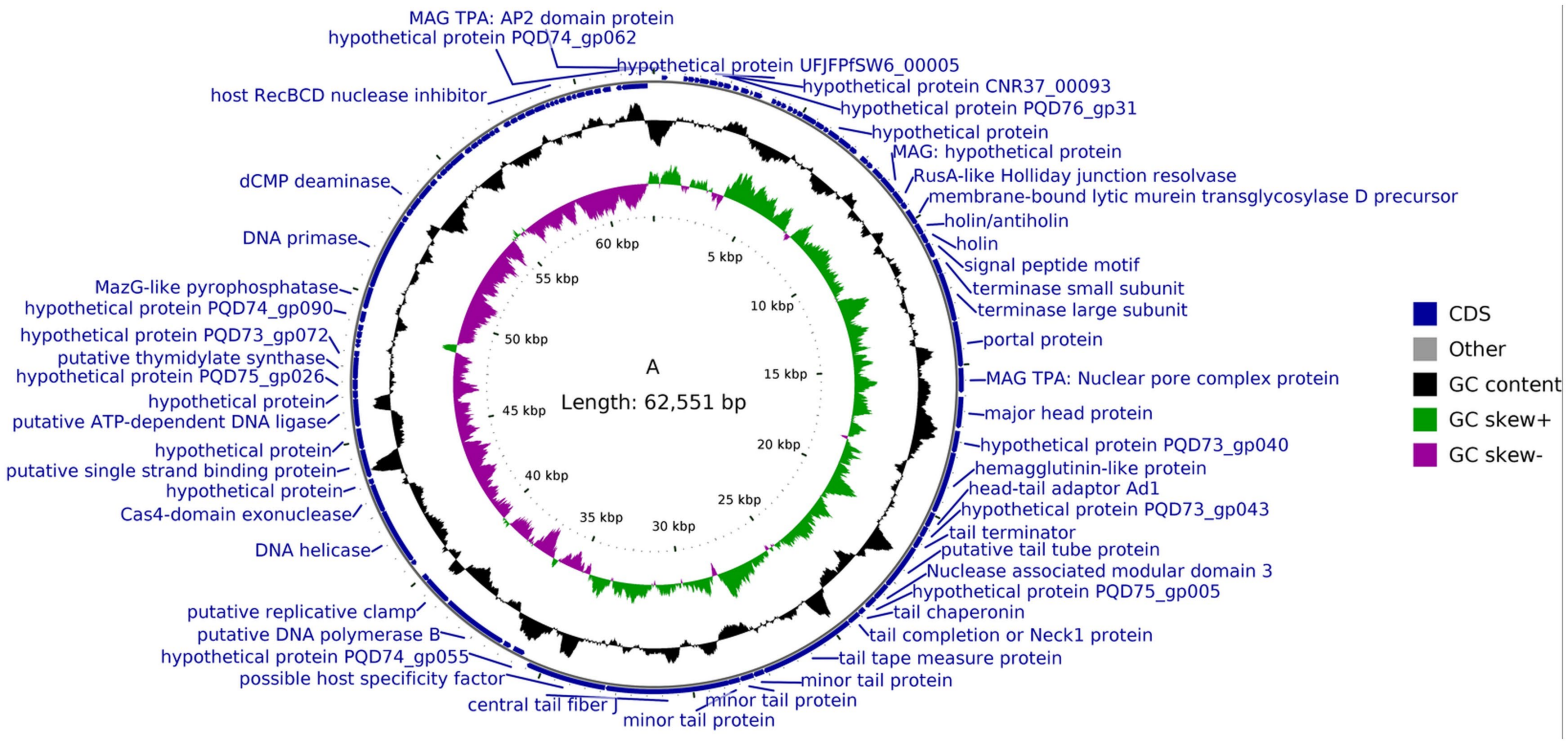
Circular genomic map of the complete genome of bacteriophage GJKY-A. The outermost circle represents the predicted protein genes of bacteriophage GJKY-A. The second outermost circle represents the distribution of the specific G + C content. The innermost circle represents GC skew, and the calculation formula is (G – C)/(G + C). Green indicates (G – C)/(G + C) > 0 and purple indicates (G – C)/(G + C) < 0.

Functionally annotated ORFs were grouped into several categories: (i) structural proteins, including portal protein (A_37), major head protein (A_39), head–tail adapter Ad1 (A_42), tail terminator protein (A_44), tail chaperone protein (A_48), tail ruler protein (A_50), and minor tail protein (A_51); (ii) DNA replication and processing enzymes, including DNA helicase (A_62) and DNA primase (A_78); and (iii) host lysis proteins, including holin (A_33) and endolysin endopeptidase (A_53). Importantly, no lysogeny-associated genes (e.g., integrase, excisionase, or repressor) were identified, indicating that GJKY-A is a strictly lytic bacteriophage (Figure 6).

### 3.4 Phylogenetic analysis of bacteriophages

The genome of phage GJKY-A was aligned to sequences in the NCBI database, demonstrating no significant nucleotide sequence similarity to any publicly available phage genomes. The genome of this phage has been submitted to the NCBI database with accession number PX310626. In addition to whole-genome comparisons, sequence similarity analyses of specific key phage protein genes such as the large terminase subunit and major capsid protein are commonly used for phage classification (Mohammed et al., 2023). The large terminase subunit is crucial for phage-driven DNA packaging and translocation, while holin is a lysis-related protein that enables phage-mediated degradation of the host cell wall and release of progeny virus particles. In phage GJKY-A, genes encoding the large terminase subunit, holin, and major head protein proteins were identified.

Based on alignment of the amino acid sequences of genes encoding the large terminase subunit of phage GJKY-A with sequences in the NCBI database showed coverage of 96% with *Stenotrophomonas phage vB_SmaS_BUCT548* and the identity was 73.69%. Similarly, the alignment of the holin protein showed sequence coverage with *Stenotrophomonas* sp. *BIO128-B* strain of 84% and identity of 41.67%. The comparison results of the major head proteins also showed that phage GJKY-A is closely related to *Stenotrophomonas phage Salva* and *Stenotrophomonas phage vB_SmaS_BUCT548* with coverage of 99% and identity of 73.24% for the former.

**Figure 7:**
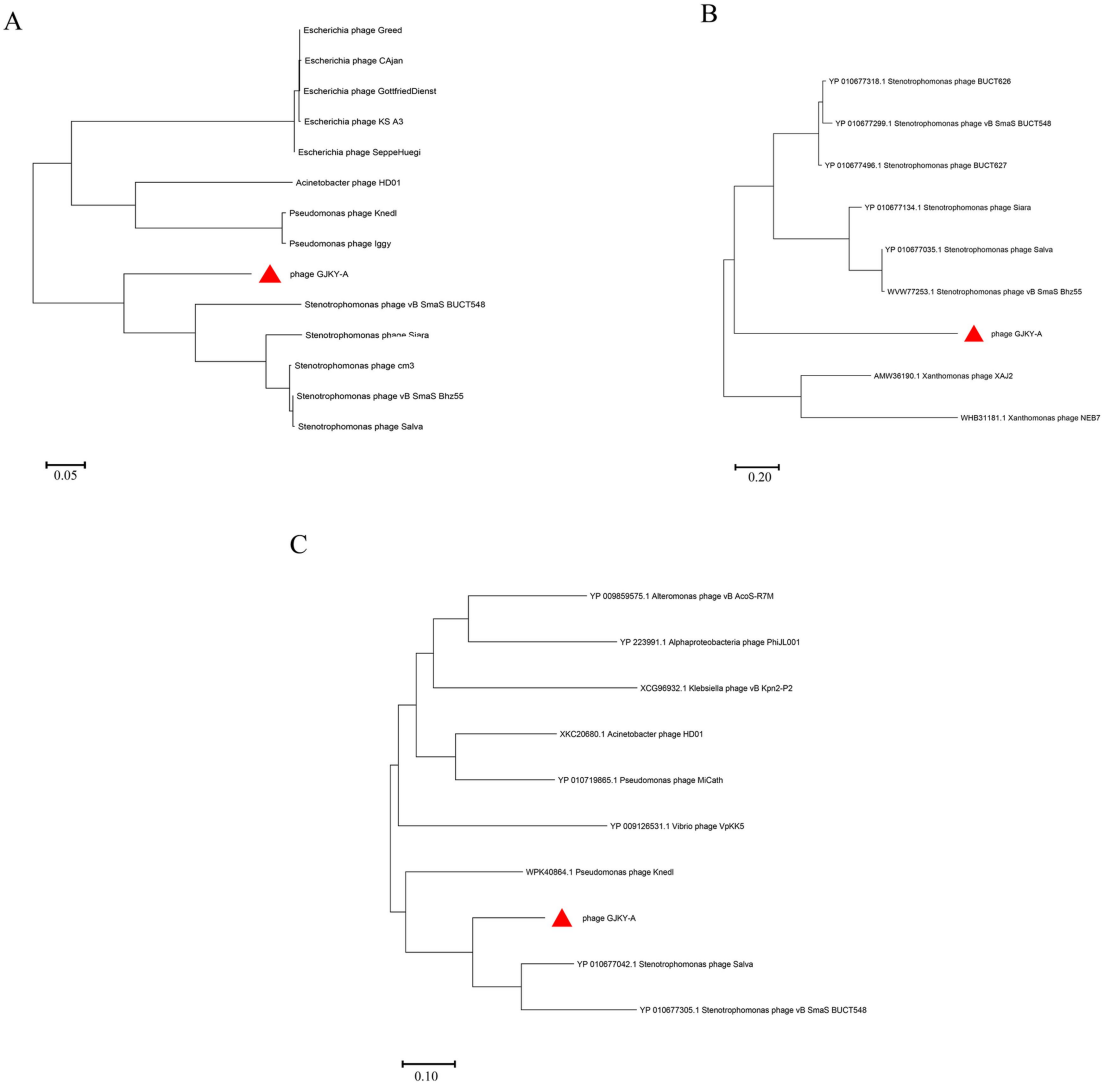
Phylogenetic trees constructed based on the amino acid sequences of specific proteins (using the maximum-likelihood method). **(A)** Large terminase subunit protein; **(B)** holin protein; **(C)** major head protein.

Selected sequences with high similarity were used to construct a phylogenetic tree with MEGA11 software based on the maximum-likelihood method. The results showed that the genes encoding the large subunit protein of the terminase and the gene encoding the perforin protein of GJKY-A belong to the same branch cluster as other phages of the genus Stenotrophomonas. Moreover, the branch lengths are all greater than those of other nodes in this cluster. This implies that these two proteins may have experienced a relatively rapid evolutionary rate or have unique functional adaptations (Suvorov and Schrider, 2024) (Figure 7). Phylogenetic analysis of the main head proteins indicated that phage GJKY-A clusters with *Stenotrophomonas phage Salva* and *Stenotrophomonas phage vB_SmaS_BUCT548*, belonging to the same branch and having a relatively close genetic relationship.

These methods allow for hierarchical clustering of phages based on their gene content and demonstrate the existence of characteristic genes that are stable across entire genera, subfamilies, or families (Turner et al., 2021). Therefore, these results confirmed that phage GJKY-A is closely related to Stenotrophomonas phages with regard to the sequences and structures of these specific proteins, suggesting that it may have retained similar functional protein sequences during evolution.

### 3.5 Inhibition of biofilm formation

**Figure 8:**
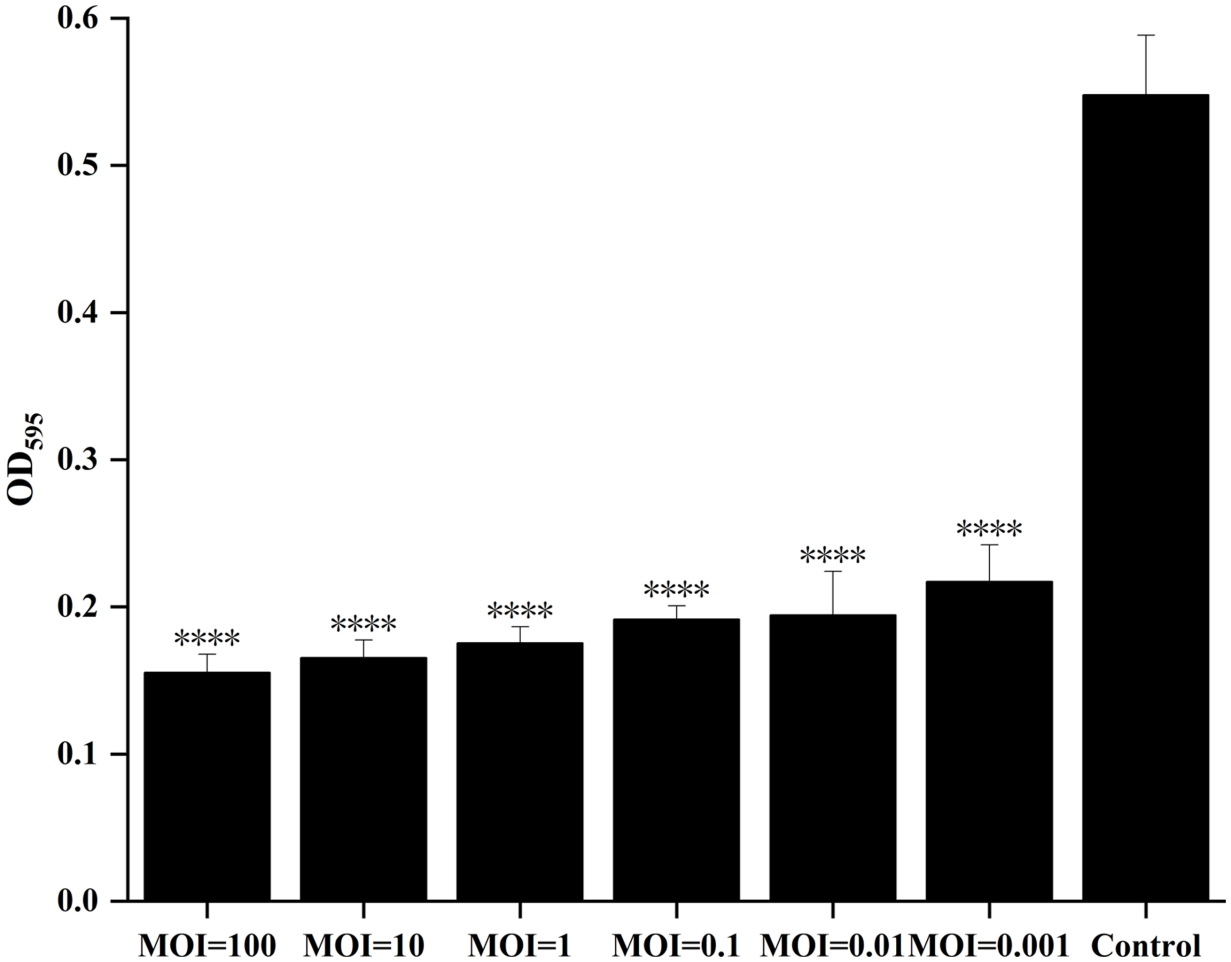
The inhibitory effect of bacteriophage GYKY_A on biofilm formation of the host bacteria after 24 h of cultivation. The results are expressed as OD595 values, indicating the degree of biofilm formation. Compared with the control group, the OD600 values of GJKY-A at different MOI levels (100, 10, 1, 0.1, 0.01, 0.001) were significantly reduced (**** indicates *p* < 0.0001).

Phage GJKY-A was co-incubated with its host bacterium at different MOIs, and the inhibitory effect on biofilm formation was evaluated using the crystal violet staining method in 96-well microtiter plates. As shown in Figure 8, compared with those of the control group, the OD595 values decreased by 71.99, 69.82, 67.99, 65.07, 64.52, and 60.38% at MOIs of 100, 10, 1, 0.1, 0.01, and 0.001, respectively. Therefore, phage GJKY-A showed a significant antibacterial effect. In addition, as the MOI value increased, the inhibitory effect gradually strengthened, indicating that the biomass of the biofilm decreased in a dose-dependent manner.

## 4 Discussion

Citrus is one of the most economically important crops worldwide, constituting the largest fruit category globally and is the most widely cultivated, economically vital fruit tree in Southern China (Guo et al., 2019). Citrus fruits are rich in vitamins, pectin, and organic acids, and exhibit antibacterial, antitumor, anti-inflammatory, and antioxidant activities (Pei et al., 2022). However, productivity and fruit quality are seriously threatened by diseases and pests—including citrus canker (*Xanthomonas citri*), huanglongbing, anthracnose, gray mold, yellow vein disease, Penicillium and Aspergillus rots, psyllids, leaf miners, and spider mites—with citrus canker being one of the most destructive bacterial diseases (Mendonça et al., 2022; Nugroho et al., 2022; Shahbaz et al., 2022). Current control measures include deployment of resistant or less susceptible cultivars, removal of infected seedlings, routine inspections and pruning, windbreak establishment, and applications of copper-based bactericides, antibiotics, and insecticides (Long et al., 2022; de Carvalho et al., 2022; Behlau et al., 2021). However, prolonged chemical use has led to soil and water contamination, pesticide residues, and the emergence of resistant bacterial populations (Carvalho et al., 2020; Pruvost et al., 2021), underscoring the need for eco-friendly, effective alternatives. Phage therapy, as a sustainable alternative, is attracting increasing attention due to its various advantages such as being green and safe, having no chemical residues, and posing a low risk of resistance development.

In this study, we isolated a phage named GJKY-A from the wastewater of a sewage treatment plant in Ji’an City, Jiangxi Province. This phage showed strong lytic activity against *Xanthomonas citri strain 3,213*. In addition, phage GJKY-A remained stable at 40–50 °C and pH 3–12, showing strong stability under different environmental conditions. This suggests that the phage can adapt to regular changes in the external environment, supporting its potential for future field control applications.

Through genomic sequencing, we found that the genome of GJKY-A comprises double-stranded DNA of 62,551 bp with a GC content of 44.81%. Functional annotation indicated that the GJKY-A genome encodes a variety of functional genes, including structural proteins, DNA replication and processing enzymes, and host lysis proteins. No genes related to lysogeny were found, suggesting that GJKY-A is a strictly virulent phage. GJKY-A exhibited a long-tailed morphology.

The large subunit of terminase is an indispensable functional protein in the phage DNA packaging process. The sequence of the large subunit of terminase is highly conserved and is therefore commonly used for phylogenetic analysis and evolutionary research (Grenyer et al., 2018). Perforin is a small hydrophobic transmembrane protein that forms transmembrane pores on the cell membrane, causing the cells to rupture. The presence or absence of perforin and its sequence characteristics can serve as an important basis for phage classification. For example, certain phages may exhibit different lytic activities and host spectra due to the specificity of perforin (Zhu et al., 2024; Pollenz et al., 2022). The sequences of the major head proteins are also relatively conserved during the evolutionary process. This conservation makes the major capsid proteins an important marker in the classification and evolutionary analysis of phages (Sugai et al., 2023). Based on these key gene products with conservatism and important functions, the large subunit of terminase, holin, and major head protein were selected to analyze the taxonomic status of phage GJKY-A. The phylogenetic trees constructed by these three proteins all showed that this phage was on the same evolutionary branch and clustered with *Stenotrophomonas phage*. This indicates that Given that the host bacterium of GJKY-A belongs to the genus Xanthomonas, this close relationship suggests the potential for cross-genus infection and can bind to the receptors of *Xanthomonas citri strain 3,213*. A previous study showed that although the phage StenR_269 was isolated from *Stenotrophomonas rhizophila*, its DNA polymerase gene clusters with *Pseudomonas phages*, indicating that taxonomic assignment should be based on functional genes rather than a single host source (Yakubovskij et al., 2023).

In addition, GJKY-A significantly inhibited the development of citrus canker lesions on leaves caused by the host bacteria (Xcc strain 3,213) in our ex vivo experiments, indicating its potential as a biological control agent. After the inoculation of bacteriophages, the number of bacteria in the leaf lesions decreased significantly, and the expansion of the lesions was significantly inhibited. In the control group without the application of bacteriophages, as the culture time extended, the lesions continued to expand and the bacteria proliferated actively, indicating that the pathogen has the ability to rapidly colonize and damage plant tissues in the absence of effective intervention (Ke et al., 2023). This comparison fully verifies the effectiveness of bacteriophages as targeted antibacterial agents.

The cell membrane inhibition experiment demonstrated that the bacteriophage GJKY-A significantly interferes with the formation of the host bacterial cell membrane. However, in the previous text, when determining the optimal MOI of phages, the optimal MOI was found to be 1, and the phage titer was not the highest at an MOI of 100. This is because the optimal MOI for phage determination and that for cell membrane inhibition are not the same. The former measures whether the virus can replicate completely and be released, while the latter only reflects whether the integrity of the cell membrane is damaged. When the MOI is 100, a single host bacterium is adsorbed by multiple phages simultaneously, the host bacterium lyses prematurely due to excessive adsorption, reducing the yield of progeny phages and thus resulting in a lower phage titer (Zou et al., 2024). However, during the inhibition of cell membrane formation, the adsorption of host bacteria by a high-density of phages will form a “hole effect.” The tail fiber proteins of phages aggregate on the cell membrane surface, destroying the integrity of the phospholipid bilayer. In addition, intracellular substances such as murein peptides and endolysins released can further interfere with the membrane synthesis of the remaining cells, playing a “synergistic destruction” role (Ding et al., 2025). As the cell membrane is a key barrier for bacteria to maintain structural integrity and to regulate substance exchange and energy metabolism, its biosynthesis process is a classic target for various antibacterial agents. The discovery that bacteriophage GJKY-A can significantly inhibit the formation of the host bacterial cell membrane reveals its potential antibacterial mechanism and provides an important basis for the development of new anti-infection strategies.

Research has shown that, the study on phages Cp1 and Cp2 of Xcc by Shahbaz et al. (2022) and the results related to phage XacN1 by Yoshikawa et al. (2018), our findings expand the understanding of the diversity of Xcc phages and their potential applications in biological control. The discovery of GJKY-A not only enhances the knowledge of Xcc phages, but its specific lytic activity against Xcc further indicates its potential as an effective biological control agent. Although this study demonstrates the high-efficiency antibacterial ability of phages under the present experimental conditions, their actual field application still faces multiple challenges. Moreover, combining phages with other biological control strategies is expected to enhance the overall prevention and control effect. For example, certain antagonistic microorganisms such as *Bacillus amyloliquefaciens* ZJLMBA1908 can directly inhibit the growth of Xcc by secreting lipopeptide antibiotics (Ke et al., 2023), while other endophytes such as *Kosakonia cowanii GN223* may enhance the host’s own defense ability through induced systemic resistance (Lai et al., 2021). Combining these biological agents with complementary mechanisms with phages may form a multi-level and synergistic green prevention and control system, reducing the dependence on traditional copper-based fungicides and antibiotics and thereby lowering the risk of environmental pollution.

## 5 Conclusion

Citrus canker has a significant economic impact on the citrus industry owing to its high transmissibility and difficulty in control. Currently, the most common management strategies rely on chemical fungicides; however, their long-term application has led to the emergence of resistant strains and soil pollution. To explore eco-friendly alternatives, a bacteriophage designated GJKY-A was isolated from sewage in this study. GJKY-A exhibited a typical long-tailed morphology, high host specificity, and high environmental stability, remaining stable at 40–50 °C and pH 3–12. The bacteriophage has a double-stranded DNA genome of 62,551 bp with a GC content of 44.81%. The phylogenetic tree constructed using conserved, key proteins indicates that this newly isolated phage shares a close phylogenetic relationship with the phages targeting the bacterial genus Stenotrophomonas. In terms of the control effect, phage GJKY-A effectively inhibited the formation and growth of the host bacteria and prevented the further development of citrus canker on citrus leaves.

In conclusion, this study confirmed the strong inhibitory potential of phage GJKY-A against *Xcc strain 3,123* under *in vitro* conditions, providing experimental evidence for the development of new environmentally friendly plant protection technologies. Further exploration is needed to determine the persistence of phages in complex agricultural ecosystems, their host specificity, and their interaction with other biological control factors. At the same time, field trials should be strengthened for verification to promote the transition of phages from the laboratory to practical applications.

## Data Availability

The datasets presented in this study can be found in online repositories. The names of the repository/repositories and accession number(s) can be found: https://www.ncbi.nlm.nih.gov/genbank/, the datasets BankIt3002511 GJKY-A PX310626.
