# Supplementary material for: Isolation, identification, and genomic analysis of bacteriophage GJKY-A for the prevention and treatment of citrus canker
Source: Front Microbiol. 2025 Dec 12;16:1703194. doi: 10.3389/fmicb.2025.1703194 (PMC12742470; doi:10.3389/fmicb.2025.1703194)
Supplement: Supplementary file 1 [file Table_1.docx]

**Supplementary Table S1.** Predictive analysis of major functional proteins encoded by open reading frames (ORFs) in the complete genome of bacteriophage GJKY_A.

| ORFs | Predicted function | Protein sequence ID |
| --- | --- | --- |
| A_1 | AP2 domain protein | DAK63659.1 |
| A_6 | hypothetical protein | WAX22719.1 |
| A_7 | hypothetical protein | ATW58300.1 |
| A_8 | hypothetical protein | YP_010677339.1 |
| A_22 | hypothetical protein | UFK09547.1 |
| A_28 | hypothetical protein | QMP83374.1 |
| A_29 | RusA-like Holliday junction resolvase | YP_010677131.1 |
| A_31 | membrane-bound lytic murein  transglycosylase D precurso | AVO24087.1 |
| A_32 | antiholin | YP_010677133.1 |
| A_33 | holin | YP_010677318.1 |
| A_34 | signal peptide motif | YP_010677036.1 |
| A_35 | terminase small subuni | YP_010677301.1 |
| A_36 | terminase large subunit | YP_010677315.1 |
| A_37 | portal protein | YP_010677303.1 |
| A_38 | Nuclear pore complex protein | DAK65937.1 |
| A_39 | major head protein | YP_010677042.1 |
| A_40 | hypothetical protein | YP_010677044.1 |
| A_41 | hemagglutinin-like protein | AMW36125.1 |
| A_42 | head-tail adaptor Ad1 | YP_010677046.1 |
| A_43 | hypothetical protein | YP_010677047.1 |
| A_44 | tail terminator | YP_010677048.1 |
| A_45 | putative tail tube protein | YP_010677404.1 |
| A_46 | Nuclease associated modular domain 3 | CAB4121893.1 |
| A_47 | hypothetical protein | YP_010677211.1 |
| A_48 | tail chaperonin | YP_010677212.1 |
| A_49 | tail completion or Neck1 protein | YP_010677213.1 |
| A_50 | tail tape measure protein | YP_010677400.1 |
| A_51 | minor tail protein | YP_010677153.1 |
| A_52 | minor tail protein | YP_010677055.1 |
| A_53 | minor tail protein/endolysin endopeptidase | YP_010677056.1 |
| A_54 | central tail fiber J | YP_239291.1 |
| A_55 | possible host specificity factor | YP_239292.1 |
| A_56 | hypothetical protein | YP_010677161.1 |
| A_58 | putative DNA polymerase B | YP_010677222.1 |
| A_59 | putative replicative clamp | YP_010677390.1 |
| A_62 | DNA helicase | YP_010677469.1 |
| A_63 | Cas4-domain exonuclease | YP_010677468.1 |
| A_64 | hypothetical protein | AMW36178.1 |
| A_65 | putative single strand binding protein | BAQ89914.1 |
| A_66 | hypothetical protein | WBQ35296.1 |
| A_67 | putative ATP-dependent DNA ligase | YP_010677382.1 |
| A_68 | hypothetical protein | BAV80881.1 |
| A_69 | hypothetical protein | YP_010677232.1 |
| A_70 | putative thymidylate synthase | YP_009603604.1 |
| A_71 | hypothetical protein | YP_010677076.1 |
| A_76 | hypothetical protein | YP_010677177.1 |
| A_77 | MazG-like pyrophosphatase | YP_010677079.1 |
| A_78 | DNA primase | YP_010677179.1 |
| A_81 | dCMP deaminase | YP_010677083.1 |
| A_101 | host RecBCD nuclease inhibitor | YP_009275481.1 |
| A_110 | hypothetical protein | YP_010677205.1 |
